# Single-center prospective experience with Optilume^®^ drug-coated balloon for recurrent urethral strictures: preliminary functional and safety outcomes

**DOI:** 10.1007/s11255-025-04922-3

**Published:** 2025-11-29

**Authors:** Antonio Cretì, Mauro Ragonese, Pierluigi Russo, Nazario Foschi, Giovanni Battista Filomena, Antonio Silvestri, Fabrizio Fantasia, Vincenzo Cavarra, Eros Scarciglia, Francesco Pio Bizzarri, Mariachiara Sighinolfi, Bernardo Maria Cesare Rocco, Francesco Pinto

**Affiliations:** 1 Department of Urology, Fondazione Policlinico Agostino Gemelli IRCSS, Largo Agostino Gemelli 1, 00167 Rome, Italy; 2 Department of Urology, C.Ur.E.-Centro Urologico Europeo, Mater Olbia Hospital, Olbia, OT Italy; 3 Department of Life Science, Health, and Health Professions, Rome, Italy

**Keywords:** Recurrent urethral stenosis, Drug coated balloon, Paclitaxel

## Abstract

**Background:**

Urethral stricture disease remains a challenging condition in urology, particularly in cases with recurrent anterior urethral narrowing following prior endoscopic treatments. Traditional options such as dilation and direct vision internal urethrotomy (DVIU) are limited by high failure rates. The Optilume^®^ drug-coated balloon (DCB) delivers mechanical dilation combined with localized paclitaxel delivery, aiming to reduce restenosis and improve durability.

**Objective:**

To evaluate the short-term functional outcomes and safety of Optilume^®^ DCB in patients with recurrent urethral strictures.

**Design, setting, and participants:**

This was a prospective single-center study including 35 male patients with anterior urethral strictures ≤ 3 cm and at least one prior endoscopic treatment. Outcomes were assessed at 1, 3, and 6 months post-procedure.

**Outcome measurements and statistical analysis:**

Primary outcomes included changes in Qmax (uroflowmetry), post-void residual (PVR), IPSS, and erectile function (IIEF-5). Recurrence was defined as symptomatic deterioration, Qmax < 10 mL/s, or need for retreatment. Paired t-tests were used for pre- and post-treatment comparisons, with p < 0.05 considered statistically significant. As this was an exploratory pilot study, no formal sample size calculation was performed; analyses were descriptive and hypothesis-generating.

**Results and limitations:**

Mean Qmax improved from 10.2 ± 4.9 to 21.6 ± 3.1 mL/s (p < 0.001), and PVR decreased from 74.6 ± 36.3 to 24.8 ± 16.0 mL (p < 0.001). IPSS improved from 21.8 ± 4.8 to 8.7 ± 2.0 (p < 0.0001), and IIEF-5 scores increased from 13.7 ± 7.7 to 18.5 ± 7.6 (p = 0.012). The recurrence rate at 6 months was 8.6% (3/35). Minor adverse events included transient hematuria and dysuria. No Clavien-Dindo grade ≥ 2 complications were recorded. Study limitations include its single-arm, non-randomized design and relatively short follow-up, limiting direct comparison with DVIU, urethroplasty, or emerging minimally invasive surgical therapies (MISTs).

**Conclusions:**

Optilume^®^ DCB treatment demonstrated significant improvements in urinary flow and symptoms with a low recurrence and complication rate at 6 months. It may serve as a minimally invasive alternative for patients unsuitable or unwilling to undergo urethroplasty. Further prospective evaluation is warranted, including its potential role in bladder neck sclerosis and benign prostatic obstruction.

## Introduction

Urethral stricture disease represents a common and recurrent challenge in male urology, resulting from progressive fibrotic remodeling of the urethral epithelium and corpus spongiosum This process, often triggered by iatrogenic trauma, external injury, infection, inflammatory conditions such as lichen sclerosus, radiation-induced ischemia and tissue damage—particularly following pelvic radiotherapy for prostate or rectal cancer—or idiopathic mechanisms, leads to luminal narrowing, increased voiding resistance, and symptomatic lower urinary tract obstruction. If left untreated, strictures may cause recurrent urinary tract infections, bladder decompensation, and upper urinary tract deterioration, ultimately impairing renal function and quality of life [1, 2].

Short-segment urethral strictures are traditionally managed with minimally invasive techniques such as dilation or direct vision internal urethrotomy (DVIU). Although technically simple and well tolerated, these approaches are limited by high recurrence rates—particularly in patients with prior endoscopic interventions—due to persistent fibroproliferative activity at the stricture site [3]. In contrast, urethroplasty remains the definitive treatment with long-term success rates exceeding 85%, yet its invasiveness, perioperative morbidity, prolonged recovery time and the steep learning curve for the surgical procedure may preclude its use [4].

In this context, the Optilume^®^ drug-coated balloon (DCB; Urotronic Inc.) has emerged as a novel therapeutic alternative that combines radial dilation with the local delivery of paclitaxel—an antiproliferative agent widely used in cardiovascular interventions to prevent restenosis. Paclitaxel acts by disrupting microtubule function and arresting fibroblast proliferation, thereby modulating the wound-healing cascade and reducing extracellular matrix deposition [5, 6]. This dual-action strategy aims to enhance the durability of endoscopic treatment while maintaining the procedural simplicity of dilation.

The efficacy and safety of the Optilume^®^ DCB has been demonstrated in different prospective trials, including the ROBUST I feasibility study and the ROBUST III randomized controlled trial, both of which reported significant improvements in urinary flow rates, symptom scores, and retreatment-free survival in patients with recurrent bulbar strictures [7, 8]. However, real-world data outside controlled trial settings remain scarce, and its effectiveness in diverse patient cohorts—including those with long-standing indwelling catheters or bladder neck involvement—requires further evaluation [9].

The objective of this study was to prospectively evaluate the short-term functional outcomes, safety, and recurrence rates following treatment with the Optilume^®^ DCB in men with recurrent anterior urethral strictures.

## Materials and methods

### Study design and population

A prospective, observational single-center study was conducted between June 2023 and January 2025. Thirty-five consecutive male patients with urethral strictures ≤ 3 cm in length, Qmax < 15 mL/s, and IPSS ≥ 13 were enrolled. Inclusion required at least one prior endoscopic treatment. Exclusion criteria included lichen sclerosus, artificial urinary sphincter, pelvic radiation, neurogenic bladder and active urinary tract infection. Two patients with bladder neck stenosis and one with penile urethral stricture were treated under compassionate off-label use after multidisciplinary evaluation and informed consent.

This study was approved by the local Institutional Review Board (IRB) and was conducted in accordance with the Declaration of Helsinki. Written informed consent was obtained from all participants prior to inclusion in the study.

In Table 1 are listed the baseline characteristics of the patients.

**Table 1:** Baseline characteristics of the patients

Characteristic	Value
Mean age (years)	63 (range 41–81)
Stricture location	Bulbar (82.9%), Penile (17.1%), Bladder neck (5.7%)
Mean stricture length (cm)	1.2 (range 0.5–3.0)
≥ 2 prior endoscopic interventions	34.3%
Median time since last intervention (months)	16 (range 6–48)
Etiology	Iatrogenic (60.0%), Idiopathic (25.7%), Traumatic (14.3%)
Comorbidities	Hypertension (45.7%), Smoking (37.1%), Diabetes (28.6%)
Indwelling urinary catheter	5 patients (14.3%)
Qmax (mL/s)	10.1 ± 4.9 (n = 30)*
IPSS	21.8 ± 4.8 (n = 35)
IIEF-5	13.7 ± 7.7 (n = 30)*

*Excludes catheterized patients where measurement not applicable

### Procedure

All procedures were performed under sedation or general anesthesia. A retrograde urethrogram and flexible cystoscopy was used to confirm stricture length and location. In patients with indwelling catheters, stricture length and site were confirmed after temporary catheter removal with retrograde urethrography and flexible cystoscopy. A pre-procedural internal urethrotomy was performed in selected cases in which it was essential to insert the balloon catheter. The Optilume^®^ DCB was advanced over a guidewire and positioned. The balloon was inflated at rated burst pressure (RBP) and maintained in situ for 7 min to ensure optimal drug delivery. Subsequently, balloon deflation and removal under vision was performed. A 14/16 Fr Foley catheter was inserted on guidewire and left in place for 24 h.

### Postoperative care

Patients were discharged without any specific therapy and were advised to maintain adequate hydration. Pain was managed with paracetamol or NSAIDs. Alpha-blockers were not routinely prescribed unless persistent voiding symptoms were reported. Patients were instructed to abstain from sexual activity for 15 days. Condom use was advised for 30 days following the procedure, and for 90 days in case of a partner of childbearing potential, to avoid potential paclitaxel exposure according to the previous indications of the different ROBUST trials [7, 8].

### Follow-up and statistical analysis

Follow-up was performed at 3 and 6 months, with uroflowmetry, post-void residual (PVR), IPSS-Qol, IIEF-5 and clinical evaluation. Recurrence was defined as a symptomatic voiding deterioration (IPSS increase ≥ 5 points), Qmax < 10 mL/s, or need for further intervention. Flexible cystoscopy was reserved for patients with symptomatic deterioration or inconclusive uroflow findings. Adverse events were graded using the Clavien-Dindo classification. Continuous variables were expressed as mean ± standard deviation (SD). Pre- and post-treatment values are compared using paired two-tailed t-tests. Subgroup analysis is conducted to compare functional outcomes in patients with and without indwelling urinary catheters at baseline. For each subgroup, changes in Qmax,IPSS and IIEF-5 were analyzed independently. Statistical significance is defined as p < 0.05. For comparison between catheterized and non-catheterized patients, independent-samples t-tests were performed. All statistical analyses are performed using SPSS version 28.0 (IBM Corp., Armonk, NY, USA).

## Results

### Functional outcomes

Of the 35 patients enrolled, all completed follow-up through 6 months. Significant improvements were noted across all primary endpoints, as depicted in Table 2.

**Table 2:** Functional Outcomes at Baseline, 3 and 6 months

Parameter	Baseline mean	3-month mean ± SD	6-month mean ± SD	Δ at 3 M	Δ at 6M	p (BL vs 3M)	p (BL vs 6M)
Qmax (mL/s)	10.2	19.5 ± 3.8	21.6 ± 3.1	+ 9.3	+ 11.4	< 0.001	< 0.001
PVR (mL)	74.6	32.0 ± 21.4	24.8 ± 16.0	−42.6	−49.8	< 0.01	< 0.01
IPSS	21.8	11.2 ± 3.6	8.7 ± 2.0	−10.6	−13.1	< 0.001	< 0.0001
IIEF-5	13.7	17.1 ± 7.2	18.5 ± 7.6	+ 3.4	+ 4.8	0.045	0.012

These functional gains were consistent across timepoints and were confirmed by patient-reported improvement in urinary symptoms and quality of life.

Among the 5 patients with long-term indwelling catheters at baseline, preoperative Qmax was not measurable due to absence of spontaneous voiding. Following treatment, 4 of these patients achieved catheter-free voiding with a mean Qmax of 13.7 mL/s. One patient continued intermittent catheterization due to suspected detrusor underactivity. In contrast, patients without catheters (n = 30) showed a significant Qmax improvement from 10.1 to 21.9 mL/s (p = 0.0011). A similar trend was observed for IPSS. IIEF-5 analysis was limited to patients without catheters at baseline to avoid potential bias.

**Fig. 1:**
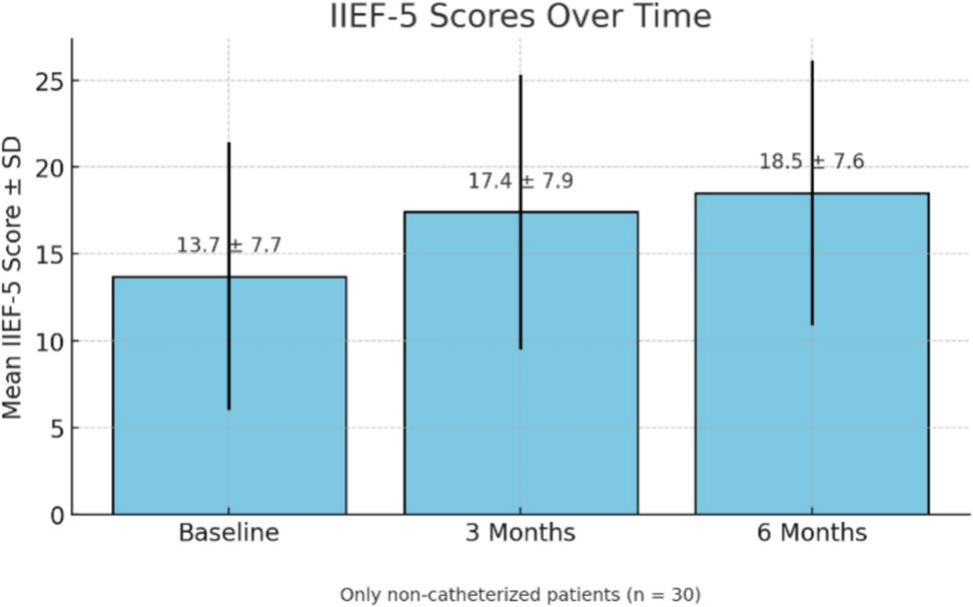
IIEF-5 scores (baseline, 3 months, 6 months). Bar chart showing mean IIEF-5 scores ± standard deviation at baseline, 3 months, and 6 months. Only non-catheterized patients (n = 30) were included to avoid bias related to indwelling catheterization. Statistical analysis was performed using paired t-tests: baseline vs 3 months (p = 0.045), baseline vs 6 months (p = 0.012)

Among the 35 patients, erectile function was assessed as a secondary outcome using the IIEF-5 questionnaire. Mean scores improved from 13.7 ± 7.7 at baseline to 18.5 ± 7.6 at 6-month follow-up (p = 0.012), as shown in Table 2 and Fig. 1. This statistically significant improvement suggests that Optilume^®^ DCB treatment does not negatively impact erectile function and may contribute to modest functional recovery in selected patients. Subgroup comparison showed that patients with indwelling catheters had a smaller improvement in Qmax (p = 0.041) but similar IPSS gains compared to non-catheterized patients.

### Safety and recurrence

No Clavien-Dindo grade ≥ 2 complications occurred. Minor adverse events included transient hematuria (n = 3), dysuria (n = 2), and one case of acute urinary retention requiring catheter reinsertion for 48 h. Recurrences occurred at 3 and 6 months, involving one distal bulbar and two proximal bulbar strictures. All were successfully retreated with Optilume^®^ DCB and remain recurrence-free at 3-month post-retreatment follow-up.”

## Discussion

Our interim findings confirm the short-term efficacy and safety of the Optilume^®^ DCB in a real-life setting. These results align closely with those of the ROBUST I feasibility trial, which—like our study—was a single-arm design without a control group, reporting comparable improvements in Qmax and IPSS at 12 months [8].

The therapeutic mechanism of Optilume^®^ DCB involves mechanical dilation to restore lumen patency and simultaneous local delivery of paclitaxel at a concentration of 3.5 µg/mm^2^. Paclitaxel acts by inhibiting smooth muscle cell proliferation and extracellular matrix deposition, two key contributors to urethral stricture recurrence. Preclinical studies and in-stent restenosis models in cardiovascular applications have confirmed the antiproliferative potential of paclitaxel [5, 6], supporting its repurposing in urological interventions.

In contrast to DVIU, which is limited by a high rate of scar reformation, and urethroplasty, which although highly effective is invasive and not suitable for all patients, Optilume^®^ offers a middle ground: a minimally invasive, outpatient-friendly procedure with improved durability [10]. It retains the ease of an endoscopic approach while addressing the biologic component of stricture recurrence.

Recent real-world studies have reinforced the effectiveness of the Optilume^®^ drug-coated balloon (DCB) in patients with complex and recurrent urethral strictures. In a multicenter Spanish cohort by Galán-Díaz et al., 156 patients with a median follow-up of 8 months showed a 73.8% treatment success rate, despite 81.4% having prior urethral manipulation [9]. No significant differences were found in the recurrence rate between patients with and without previous urethral manipulation (UD, EIU, or urethroplasty), 26.7% vs. 24%, p = 0.783.

Similarly, VanDyke et al. evaluated 122 patients, including 33 (27%) with prior failed urethroplasty. Despite a higher number of prior interventions in this group (median 3 vs. 1), success rates remained high—80.0% versus 88.9%—with no significant difference (p = 0.338). These findings underscore the utility of paclitaxel-based DCB therapy in patients with prior failed interventions, including those with a history of urethroplasty [11].

In our cohort, the presence of indwelling urinary catheters at baseline was associated with less pronounced Qmax improvement, although the majority of these patients still achieved catheter-free voiding. This may reflect more extensive fibrotic remodeling or detrusor underactivity, two common sequelae in chronically catheterized patients.

Two patients in our study with bladder neck stenosis post-TURP were successfully treated with Optilume^®^. Although this indication remains off-label, these cases support the technical feasibility and safety of DCB use at the bladder neck [12]. The use of a larger-diameter balloon may further enhance efficacy by optimizing drug penetration and mechanical remodeling, though this hypothesis warrants prospective validation. The ability to deliver paclitaxel to the fibrotic bladder outlet without thermal or incisional damage could provide an alternative to transurethral resection, especially in patients with high surgical risk.

The Optilume^®^ platform has also generated interest in the treatment of benign prostatic hyperplasia (BPH). The ongoing EVEREST study investigates the safety and efficacy of paclitaxel-coated balloon dilation in the prostatic urethra, aiming to offer symptom relief without recurring to common endoscopic techniques (TURP, laser endoscopic enucleation of the prostate). Preclinical models suggest that localized drug delivery in BPH may attenuate glandular hyperplasia and preserve flow dynamics [13].

Given the increasing role of minimally invasive surgical therapies (MISTs) in the management of both urethral stricture and BPH, a direct comparison of Optilume® DCB with alternative MISTs such as TURP, Rezūm, or laser-based approaches is warranted. Comparative trials may help define the specific subset of patients most likely to benefit from DCB, balancing efficacy, invasiveness, and durability.

Despite the encouraging outcomes, our study has some limitations. It lacks a randomized comparator arm (e.g., DVIU or urethroplasty), which restricts the strength of inference regarding relative efficacy. The sample size is modest, and the follow-up duration is limited to 6 months. Furthermore, the inclusion of off-label cases introduces heterogeneity, although it may better reflect clinical practice. Future research will aim to include a control group to strengthen comparative inference and expand data on off-label anatomical sites.

## Conclusions

This prospective single-center experience demonstrates that the Optilume^®^ DCB is a safe and effective option for the management of recurrent urethral strictures. The device yielded significant improvements in Qmax, PVR, IPSS Qol and IIEF-5 with a low complication and retreatment rate at 6 months. Its minimally invasive nature and short procedural time make it an attractive alternative in patients with recurrence after prior endoscopic treatments or in those unfit for urethroplasty.

The successful treatment of selected cases of bladder neck sclerosis, as well as ongoing investigation into its role in benign prostatic hyperplasia (BPH), suggests broader potential indications for the Optilume^®^ platform. The ability to achieve mechanical dilation combined with local paclitaxel delivery may prove advantageous in reducing fibrotic remodeling across a range of obstructive urological conditions.

Given the expanding spectrum of minimally invasive surgical therapies (MISTs), further comparative studies are essential not only in terms of efficacy and durability, but also in patient-centered outcomes such as recovery time, quality of life, and retreatment burden.

## Data Availability

No datasets were generated or analysed during the current study.
